# The impact of timing and type of donation decision on charitable giving

**DOI:** 10.1007/s00355-024-01572-9

**Published:** 2025-01-31

**Authors:** Marina Chugunova, Andreas Nicklisch, Kai-Uwe Schnapp

**Affiliations:** 1 Max Planck Institute for Innovation and Competition, Munich, Germany; 2 University of Applied Sciences of the Grisons, Chur, Switzerland; 3 Department of Social Sciences, University of Hamburg, Hamburg, Germany

## Abstract

We study how the timing and the type of donation decisions affect charitable giving. In an online real-effort experiment with a subsistence income constraint, participants could donate to a charity either before or after they worked to generate income. We find no evidence that timing affects the propensity to donate or the amounts donated. However, if a donation is expressed as a share of future income rather than an absolute amount, more people donate and they donate larger amounts. Advance commitment does not appear to have a motivational effect on effort. Our findings suggest that requesting donations as a share of future income may enhance charitable giving.

## Introduction

Inequality can cause severe social, economic, and political tensions in a society (e.g., Piketty 2014; Staab and Nachtwey 2016). It limits economic prosperity and spurs societal division as well as violent conflicts (Stiglitz 2012). Hence, curbing inequality is an important societal goal. In part, inequalities can be reduced through voluntary transfers. In 2022, total donations by US individuals amounted to 319 billion dollars, or 1.2% of the US GDP (Giving USA 2023). Reflecting the importance of charitable giving, a vast literature is dedicated to the understanding why people donate and the factors that affect giving (e.g., Andreoni 1989; Engel 2011; Dana et al. 2007; Eckel and Grossman 2003; Erkal et al. 2011).

In this paper, we consider the timing of donations, a factor that has received only limited attention in the literature so far. Many donations are made after income has been earned, but there are also instances of advance commitments to giving, that is, giving before income is earned. Examples range from religiously mandated giving such as tithing in Catholicism or zakat in Islam, to entrepreneurial initiatives,^1^ to planned giving and charitable checking accounts. In this context, our study analyzes whether the timing of donations (commitment before or after income is earned) affects the number of donors and the amounts donated. Furthermore, we ask what effect the timing of a donation has on effort.

The timing of donations may affect willingness to donate and the amounts donated. Donating before production comes with uncertainty about the size of the income (i.e., the donor does not know how much she will earn). This uncertainty is particularly important if the donor has a subsistence income threshold below which she cannot fall. If this is the case, committing to donate before own earnings are known increases the likelihood that this subsistence level will not be reached. When donating after production, this uncertainty about the income is resolved. Yet, having invested effort already, potential donors may feel a sense of entitlement to their earnings and thus be less willing to give at all or give less.

Donations can be expressed either as a share of one’s income (e.g., the tithe in Catholicism) or as an absolute amount (e.g., many nonprofit organizations enable monthly donations of a certain amount). This difference in the way donations are framed may affect people’s decision to extend effort and ultimately their decision to donate, especially when donation decisions are made before income is earned. The timing of a donation and its framing as a fixed amount or a share of the income generate a puzzling interplay of incentives that has also been under-explored so far.

In this study, we investigate how the timing and the type of donation decisions affect the propensity to donate and the amounts donated. To do so, we conduct an online experiment with the following basic structure (see details in Sect. 2). Participants are invited to decode strings of digits into corresponding letters for 3 min. They receive a piece rate for each correctly decoded string. After completing this first task, participants are informed that they will continue working on the same task again, but can now donate a part of their forthcoming earnings to the British Red Cross. In the two treatment conditions – *Ante Share* and *Ante Absolute* – participants commit to giving before doing the second decoding task and express their donations as a share or an absolute amount respectively. In the *Post* treatment condition, participants decide whether and how much they want to donate after they finish the second decoding task. We introduce the income constraint through a bonus: participants whose earnings net off donations meet the threshold receive a bonus payment. The bonus payment mimics the earnings participants could make in future “periods” if their subsistence is met.

Overall, we find no significant evidence that the timing of a donation decision relative to production affects the propensity to donate or the amount donated, both on average and conditional on the decision to donate non-zero amounts. However, when committing to donate from future earnings, framing the donation as a share rather than an absolute amount has a positive effect both on the propensity to donate and the amounts donated. Additional proxies allow us to test for the role of risk and entitlement towards one’s earnings for these decisions. We find some suggestive, but not robust evidence that both motives influence giving. Yet, in our setting, both seem to cancel each other out.

Commitment in the Ante treatments does also not appear to have a motivational effect on effort. Controlling for initial ability, however, we find that donors perform better than non-donors. Yet, we have to stress that the decision to donate is endogenous and, therefore, our experimental design does not allow us to establish a causal link between the donation decision and the effort: people may give a lot because they produce a lot, or vice versa. Finally, in the Ante treatments, we consider the difference between the actual donation decision and a *hypothetical* donation decision after income has been generated and uncertainty about passing the threshold is resolved. We find “positive” reneging: more people want to donate and the average donation amounts are revised slightly upwards.

Our paper relates to studies on the role of inter-temporal choices for charitable giving. Making a decision to donate now or in the future may affect giving due to present bias (Kölle and Wenner 2021) or due to costly self-control within the dual system of decision-making (Dreber et al. 2016; Fromell et al. 2020). Several studies focus on separating the donation pledge from the actual donation in time. In a field experiment, Breman (2011) finds that such separation increases giving. Andreoni and Serra-Garcia (2021b) show theoretically and empirically that social image concerns amplify the increase in giving when the pledge and the actual donation are not simultaneous. Kellner et al. (2019) study conditional commitments to give from a future lottery and show that participants are more likely to donate and donate more from potential future lottery winnings as compared to when they have already won. Building on this, we focus on unconditional and binding commitments to donate from earnings as a prevalent source of income for many. The fact that earnings are generated through effort is important as this may trigger a sense of entitlement towards one’s earnings (Cherry et al. 2002; Durante et al. 2014).^2^ By considering the potential motivational effects of advance commitments for production, our study also extends the literature on the role of prosocial motivations for effort provision (Imas 2014; Tonin and Vlassopoulos 2015; Kajackaite and Sliwka 2017).

The paper proceeds as follows. Section 2 presents our experimental design and develops the hypotheses. Section 3 reports the results. Section 4 contains the discussion and conclusions.

## Experimental design, hypotheses, and procedures

### Experimental design

**First investment game** After consenting to take part in the study, participants play an investment game (Gneezy and Potters 1997) to measure their risk attitudes. They receive an endowment of £0.20 and may invest any share of this endowment in a risky project. With a 50% chance, they receive 2.5 times what they invested; otherwise, their investment is lost. They also keep the money they did not invest. More risk-seeking participants should tend to invest more in the risky project.

**Fig. 1:**
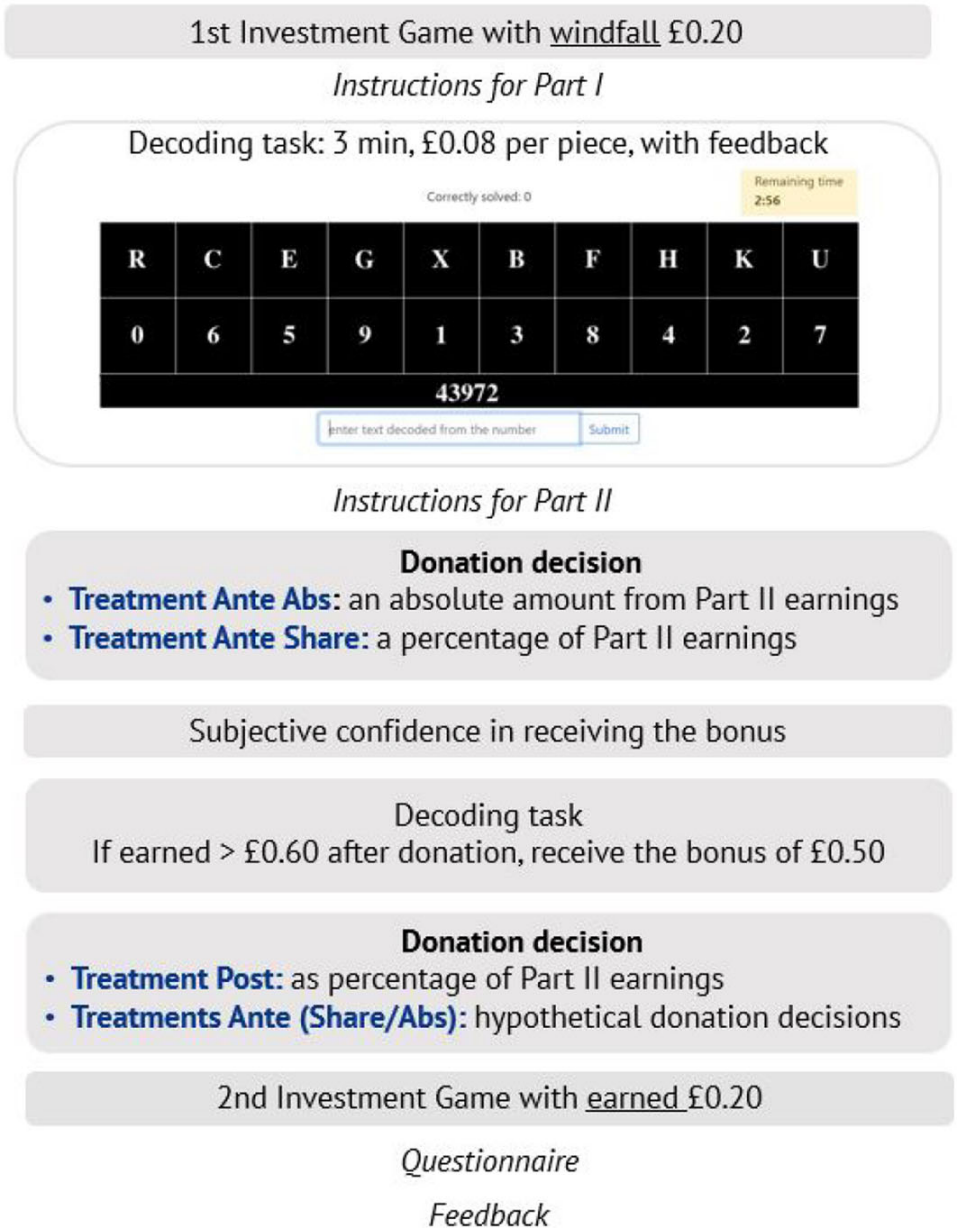
Overview of the experiment

**Part I: Decoding** After the first investment game, participants receive instructions for Part I of the experiment. They have three minutes to decode strings of five digits into corresponding letters (Erkal et al. 2011). For each correct entry, they receive £0.08. Once they decode a string correctly, they see a new string of five digits. The key to decode the string changes with every new string. On the screen, participants see the timer and the counter of how many strings they have decoded so far (see Fig. 1). At the end of Part I they learn how many strings they decoded correctly in total. Participants are free to decode as many strings as they wish in the time given. As the experiment is conducted online, participants have a natural leisure option of browsing the internet or doing anything else. Participants answer mandatory attention questions before starting with the task.

**Part II: Treatment and donation decision** Next, participants receive instructions for Part II. Part II involves another three minutes of the decoding task, but this time participants can donate parts of their earnings from Part II to the British Red Cross. The donation amounts are matched by the experimenters and then transferred to the charity. Participants are informed that they will receive a confirmation of the donation via direct messages in Prolific.

If participants earn more than £0.60 in Part II after the donations are deducted, they receive a bonus of £0.50. This threshold corresponds to about 60% of what participants earn in Part I on average (£0.95), thus offering room for reducing effort and nevertheless receiving the bonus.

**Treatments** Between the three treatments, we vary the timing and the type of donation. In the two versions of the *Ante* treatment, participants have to commit to the donation prior to the decoding task in Part II.^3^ In *Ante Share*, they express how much they want to donate as a share of their yet-to-be-obtained earnings from Part II. In this treatment, how much is actually donated to charity is a combination of the chosen share and the number of strings the participant decodes in Part II. In *Ante Absolute (Ante Abs)*, participants express how much they want to donate as an absolute amount. If they commit to donate more than their actual earnings in Part II, all of their earnings in Part II will be donated.^4^ In the *Post* treatment, participants first perform the decoding task for three minutes, receive feedback on the number of correctly decoded strings, and only then decide on how much to donate. They are asked to express their donation as a share of their income but are offered a calculator button to compute what this share means in absolute numbers.^5^

**Additional measures** In all the treatments, before performing the decoding task (and after choosing the donation in the Ante treatments), we ask participants to rate their perceived chances of meeting the income threshold and receiving the bonus on a scale from 0 to 100, where 100 represents the highest confidence. Additionally, in the Ante treatments, after completing the decoding in Part II and learning their earnings from it, participants are asked if and by how much they would like to change their donation. This question is hypothetical and does not affect their earnings.

**Second investment game** The experiment concludes with a second investment game, in which participants can use up to £0.20 *out of their earnings* from the experiment to invest under the same conditions as in the first investment game (50% chance of winning 2.5 times the investment). We use the difference between the investment with the windfall and earned endowment as a proxy for the strength of the perception of being entitled to the money earned. Larger differences indicate a stronger perception of entitlement. Participants learn the outcomes of both investment games at the end of the experiment. Both decisions are payoff-relevant.

**Postexperimental questionnaire and feedback** In the postexperimental questionnaire participants answer questions about a variety of factors that may have affected their behavior in the study: their age, gender, and employment status; trust, prosociality, risk-taking, and attitudes toward redistribution; whether they had heard of the British Red Cross before and whether they thought the money would be well spent; and whether they have worked harder because they could donate to the charity. Finally, we ask participants for their expected payoff for a study that lasts ten minutes and provide feedback on their actual earnings for all parts of the study. Figure 1 provides an overview of the experiment.

### Hypotheses

Let us assume that subject *i* distributes her income $x_i$ in a given period of time between herself and another receiver *j* (in our design *j* is the charity). *i* completes the period “successfully” if her income after the transfer to *j* meets some minimum requirements and she obtains an additional income $\hat{x}_i$.^6^ If *i* completes the period “unsuccessfully” she does not obtain an additional income $\hat{x}_i$.

Our expectations regarding the behavior of *i* are guided by a simple additive utility model $U_i(y)$ that combines purely selfish and social motives of player *i* who may transfer *y* to *j*. Each motive is embedded in one of two terms: the first one covers *i*’s own income and the second one the concave utility drawn from donating money to *j*. That is,

1 $$\begin{aligned} U_i(y)=\left( \alpha _i+Ie_i\right) \left( x_i-y+p(y)\hat{x}_i\right) +\beta _i g(y), \end{aligned}$$

with $\alpha _{i}>0$ and $\beta _{i}\ge 0$ (and typically $\beta _{i}<\alpha _i$) being *i*’s taste parameters for the selfish and the social component, while *g*(.) is a concave function. *I* is an indicator with a value of one after the income has been generated, and zero otherwise, while $e_i>0$ is another constant parameter. $e_i$ embeds the entitlement effect into the utility function: the value of the selfish component is larger after income generation than before. Finally, $p(y)\hat{x}_i$ is the expected additional earning of *i* conditional on the successful completion of the given period of time. *p*(.) is the probability of *i*’s success net of transfers. $y=0$ maximizes this probability, whereas it minimizes the social utility component.

For the optimal transfer $y^*$, the first order condition applies (i.e., $\frac{\partial U_i(y)}{\partial y}=0$), such that $y^*$ is implicitly defined as^7^

2 $$\begin{aligned} 1+|p'(y^*)\hat{x}_i|=\frac{\beta _i}{\alpha _i+Ie_i} g'(y^*). \end{aligned}$$

That is, the transfer is optimal if the marginal selfish utility in terms of current and expected additional income equals the relative (i.e., $\frac{\beta _i}{\alpha _i+Ie_i}$) marginal social utility.

There are three factors that influence the optimal $y^*$ in Eq. (2): (i) all else equal, if donations are determined after income has been generated, the entitlement effect makes the selfish utility component in Eq. (2) larger than if they are determined before. This implies that the right-hand side of the Eq. (2) is smaller. Therefore, optimal donations are smaller in the former case than in the latter.

(ii) If donations are determined after income has been generated, *i* can adjust *y* such that $p(y)=1$ (provided that *i*’s income prior to transfers is sufficiently high to complete her current period successfully). That is, $p'(y)=0$ if donations are determined after income has been generated. Provided that the social utility is sufficiently high, *i* simply sets *y* such that with her remaining income she meets the income threshold. If *i* determines the donation before income is generated, the left-hand side of the Eq. (2) increases in comparison to the former case implying that the the optimal $y^*$ in Eq. (2) decreases. That is, *i* lowers *y* to keep *p*(*y*) sufficiently high. Hence, if $x_i$ is fixed, donations are at most as large in the Ante treatments as in the Post treatment.

(iii) Finally, the threat of not passing the threshold may spur the productivity of donors. That is, the risk of not meeting the threshold that comes with the commitment in advance may motivate the donors to work harder. It appears that this effect might be particularly high if the donation is expressed in absolute amounts versus a share of the income, since in the latter setting donors still earn at least a fraction from each token of the income. In turn, donations expressed in absolute amounts may amplify the need to increase effort as producing initially does not lower the gap between one’s own income and the threshold (until the absolute amount of the donation is earned).

A priori, it seems rather difficult to predict whether donating before or after yields higher shares of donors and donations. (ii) suggests that $y^*$ solving (2) is larger in Post than in the Ante conditions. At the same time, according to (i) $y^*$ decreases in *Post* compared with *Ante*. The specific $y^*$ depends on the subjective perception of risk and entitlement for earned income. Finally, factor (iii) spurs production, from our point of view most likely in *Ante*. Particularly, we assume that the productivity effect is stronger if the donation is expressed as an absolute amount versus a share of the income.

Based on the arguments above, we hypothesize^8^:

*Hypothesis 1:* $$y^{*Ante Abs}> y^{*Ante Share}> y^{*Post}$$.

*Hypothesis 2:* $$x_i^{Ante}>x_i^{Post}$$.

*Hypothesis 3:* $$x_i^{Ante Abs}>x_i^{Ante Share}$$.

That is, we assume that the production effect is stronger in Ante than in Post, and the strongest in Ante Abs (Hypotheses 2 and 3), while the risk and the entitlement effects are confounding. Therefore, we predict that donations are the highest in Ante Abs and the lowest in Post (Hypothesis 1).

### Procedure

We collected 437 observations in an online experiment via Prolific.^9^ 147 were randomly assigned to Ante Share, 144 to Ante Absolute, and 146 to Post. Participants were residents of the UK between 25 and 65 years old, whose first language was English, and who had a platform approval rate above 90. The sample was gender-balanced. Participants were on average 41 years old.

We implemented the experiment in oTree (Chen et al. 2016). The average duration of the study was 13 min. Participants received £1.10 as a flat participation payment and on average £2.7 of an additional payment based on their performance, investment, and donation decisions.^10^ 54% were employed full-time, 17.2% were unemployed, remaining employed part-time or self-employed in equal shares.

99.5% had heard about the benefiting charity, the British Red Cross, and only about 10% of the participants expressed doubts that the money transferred to this charity would be well spent (answers 1-3 on the 7-point scale), 74.6% agreed or strongly agreed that the money would be well spent (answers 5-7 on the 7-point scale).

Participants solved 11.8 strings on average in the first decoding task. Their performance did not differ significantly by treatment (Kruskal-Wallis equality-of-populations rank test, *p*=0.17).

## Results

First, we consider whether the timing and the type of donation decisions affect the likelihood of a donation and its amount. We then proceed to consider the role of entitlement and risk in donation decisions. We conclude by analyzing individual efforts.

**Fig. 2:**
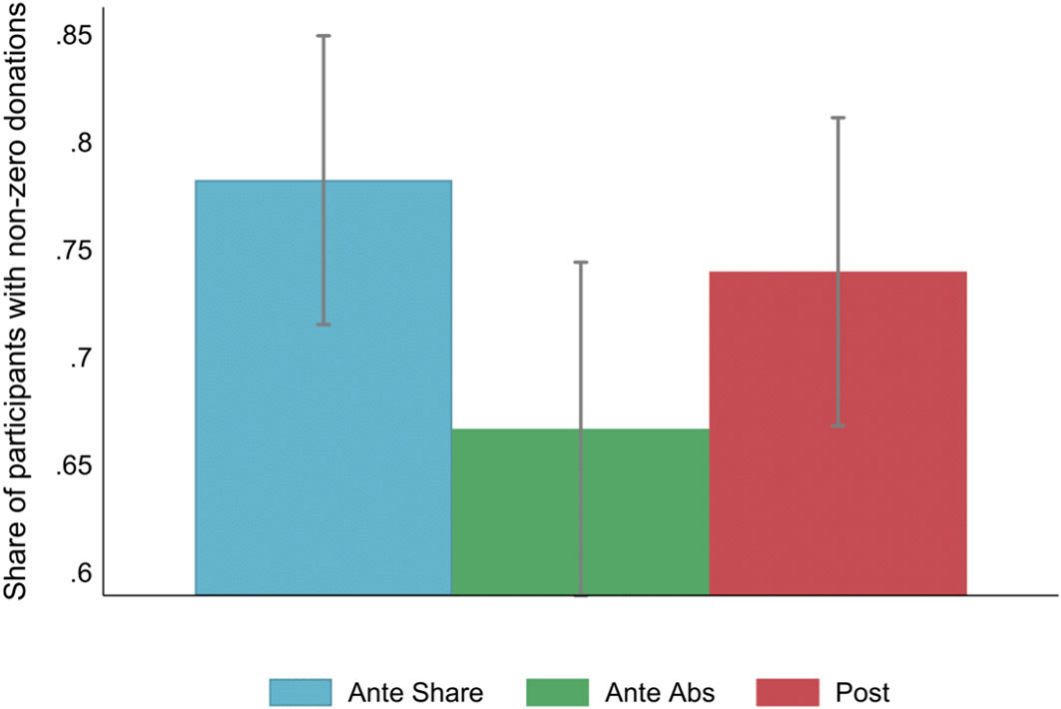
Share of participants who donated non-zero amounts by treatment. 95% confidence intervals

**Share of donors** Our data offers no evidence that the timing of the donation relative to production affects the share of people who decided to donate non-zero amounts. However, it appears that committing to donate a share of income compared to an absolute amount increases the likelihood of becoming a donor. In Ante Share, 78.2% of the participants decided to donate a non-zero amount, and about the same share (74%) decided to donate in Post (two-sample test of proportions,^11^$p=0.4$). However, only 67% chose to donate in Ante Absolute (as compared to Ante Share, two-sample test of proportions, $p=0.03$). Figure 2 illustrates this result. We reconfirm this result and run a probit regression additionally controlling for the measure of risk-seeking (the amount invested in a risky project in the first investment game), entitlement to earnings measured as a difference between an investment with the windfall and earned money, performance in the previous part (i.e., Part I in the Ante treatments and Part II in the Post treatment respectively), a measure of prosociality, the belief that the money will be well spent, and participant’s income from the experiment. Specification 1 in Table 1 presents marginal effects of the probit regression and shows that being assigned to the Ante Abs treatment reduces the probability of donating by 8.8 percentage points ($p=0.06$), holding other factors constant. Their risk preferences and entitlement toward earned income do not appear to affect this decision. Providing a sanity check, participants who report being more prosocial and believe the donated money will be well spent are more likely to become donors. Participants with higher earning expectations are less likely to donate non-zero amounts.

**Result 1:** Against our Hypothesis 1, we find no evidence that the timing of the donation decision affects the propensity to donate non-zero amounts. However, expressing the donation as an absolute amount versus a share of income appears to decrease the propensity to become a donor.

**Table 1:** Specification 1 reports the marginal effects of the probit regression where the dependent variable is a binary variable indicating non-zero donations

	(1)	(2)	(3)	(4)
Variables	Non-Zero Donation	Donation	Donation	Donation
	(marginal effects)	amount	amount	amount
				(conditional)
Ante Absolute	$-$0.088*	$-$0.105***	$-$0.093***	$-$0.088**
	(0.046)	(0.038)	(0.035)	(0.041)
Post	$-$0.020	$-$0.028	$-$0.055	$-$0.055
	(0.046)	(0.042)	(0.039)	(0.045)
Risk-seeking	$-$0.321	0.640**	0.336	0.720*
	(0.369)	(0.318)	(0.279)	(0.376)
Entitlement	$-$0.166	$-$0.650**	$-$0.281	$-$0.379
	(0.364)	(0.256)	(0.263)	(0.335)
Previous performance	0.005		0.021***	0.026***
	(0.006)		(0.005)	(0.005)
Prosocial	0.027***		0.027***	0.027***
	(0.008)		(0.006)	(0.008)
Well used money	0.099***		0.068***	0.053***
	(0.010)		(0.009)	(0.014)
Expected pay	$-$0.060***		$-$0.033**	$-$0.028
	(0.020)		(0.014)	(0.025)
Constant		0.254***	$-$0.459***	$-$0.416***
		(0.052)	(0.087)	(0.127)
Observations	437	437	437	319
R^2^	(Pseudo) 0.200	0.029	0.218	0.157

Specifications 2-4 are OLS regressions. Specification 4 is conditional on donating a non-zero amount. *Risk-seeking* is measured through the investment in the risky project in the first investment game. *Entitlement* is measured through the difference in investments between the first and the second investment games. *Previous performance* refers to the number of correct entries in Part I in the Ante treatments and in Part II in the Post treatment. *Prosociality* and *Well-used money* were reported on 10-point and 7-point scales respectively with higher values indicating higher prosociality and stronger agreement that the money will be well spent

Standard errors in parentheses

*** p<0.01, ** p<0.05, * p<0.1

**Donation amounts** The pattern is reproduced for the analysis of the amounts donated: On average, in Ante Abs, participants donated £0.25, which is significantly less than £0.35 in Ante Share (t-test, $p<0.01$). The difference in donation amounts compared to Post is marginally significant (£0.32, t-test $p=0.07$).^12^ The regression results reported in Table 1 (specifications 2–3) qualitatively reconfirm this result.

As the difference in average donation amounts may be driven by the general propensity of becoming a donor alone, we additionally estimate the donation amounts conditional on being a donor (Table 1, specification 4). It documents that donors in the Ante Absolute treatment donate about £0.09 less than in Ante Share ($p=0.03$).

It is relevant to note that the income threshold is expressed in absolute terms in all treatments. Hence, some part of the treatment difference in the donation amounts may be explained by the following mechanism: First, when committing to donate a share of income it may be slightly more computationally difficult to target a specific donation amount. Second, the donation amount is a function of the chosen share and the effort extended. Therefore, if participants have a target donation in mind but, for example, underestimate their performance improvement from Part I to II, they may donate more than they intended to donate (see the discussion on reneging below). The different framing of the donation and the threshold is unlikely to impact the propensity to donate as such but may affect the amounts donated among those who decided to donate non-zero amounts.

**Result 2:** In contrast to our Hypothesis 1, there is no significant difference between donation amounts in Ante Share and Post. Yet, participants in Ante Absolute donate significantly less than in Ante Share. In addition to the smaller share of donors in Ante Absolute, those who decide to donate, donate less.

**Role of entitlement and risk** We hypothesized that the decision to give and how much to give will be affected by the risk attitudes and the difference in entitlement triggered by considering future versus already realized earnings. We measure risk-seeking through the amount the participant allocated to a risky project in the first investment game (Gneezy and Potters 1997). To measure the difference in entitlement, we used the difference between the decision in the investment game with windfall money and with earned money, with a positive number showing that the participant invested more in the risky project from the windfall endowment. Both measures were included as controls in the regressions (variables *entitlement* and *risk-seeking*). In specification 2, in line with our reasoning, we find that more risk-seeking participants tend to donate more and participants feeling more entitled to their earnings donate less. The magnitude of the coefficients is strikingly similar. This may suggest that the two effects are equally strong and cancel each other out. However, this finding is not robust to including additional controls and only considering donors (specifications 3–4).^13^

We further explore whether there is suggestive evidence that entitlement may have differed by treatment. For that, we consider how much more participants could potentially have donated in addition.^14^ In Post, participants could have donated an *additional* £0.46, in Ante Share £0.34 (t-test, Ante Share versus Post $p=0.05$), and in Ante Abs £0.27 (t-test, Ante Abs versus Post $p<0.001$) while clearing the threshold.^15^ The finding that participants in Post donate significantly less of their “spare” money despite them knowing that they passed the threshold is consistent with them being more attached to their earnings. At the same time, the average income necessary to clear the threshold (i.e., the threshold plus donations) is higher in Ante Share and Ante Abs as compared to Post. The average necessary income corresponded to earnings from 10.8 tasks in Ante Share, 10.7 tasks in Ante Abs and 9.9 tasks in Post (t-test, Ante Share versus Post, $p=0.04$; Ante Abs versus Post, $p=0.07$, Ante Share versus Ante Abs, $p=0.8$).

**Result 3:** Our data offers directional support to a positive effect of risk-seeking and a negative effect of entitlement on donation amounts.

**Reneging** In our experiment the commitment to giving was binding. However, in the Ante treatments, we allowed participants to hypothetically reconsider their giving decision after the decoding task in Part II was finished. Hence, we can speculate on potential reneging behavior. Unexpectedly, we documented “positive” reneging: more people wanted to donate and the average donation amount in the hypothetical donation question was slightly higher than the real donation. 20% of the non-donors in the Ante treatments reported that they would have donated. The average share of hypothetical donors in both Ante treatments was 77.0% as compared to 72.5% of actual donors (t-test, $p=0.003$). The increase appears to be driven largely by the Ante Abs treatment (from 66.7% to 72.9%, t-test, $p=0.01$). The increase in Ante Share is not statistically significant (from 78.2% to 80.9%, t-test, $p=0.1$).^16^ On average, participants in Ante Share would like to donate 2.7% more, and participants in Ante Abs would like to donate an extra £0.06 (comparison between the initial and hypothetical donations, t-test, $p=0.03$ in Ante Share and $p<0.001$ in Ante Abs). However, even when considering the hypothetical donation decisions, the amounts donated in Ante Abs remain marginally lower than in Ante Share (£0.38 in Ante Share, £0.32 in Ante Abs (t-test, $p=0.08$), £0.32 in Post (t-test, $p=0.15$)).

**Result 4:** In a hypothetical question, we document “positive” reneging: more people expressed the intention to donate and the average donation amount was slightly higher.

**Effort** The commitment to giving in combination with the threshold may have generated a difference in effort extended between treatments. In Part II, participants on average decoded 1.8 additional strings as compared to Part I. In Table 2, we report the results of an OLS regression where the dependent variable is the number of strings decoded in Part II. We control for any initial differences in ability (variable *correct Part I*) and, as in the previous analysis, for risk attitudes, entitlement towards own earnings, levels of prosociality, and beliefs that money will be well spent.^17^ The regression results suggest that there are no treatment differences in the number of correctly decoded strings.

**Fig. 3:**
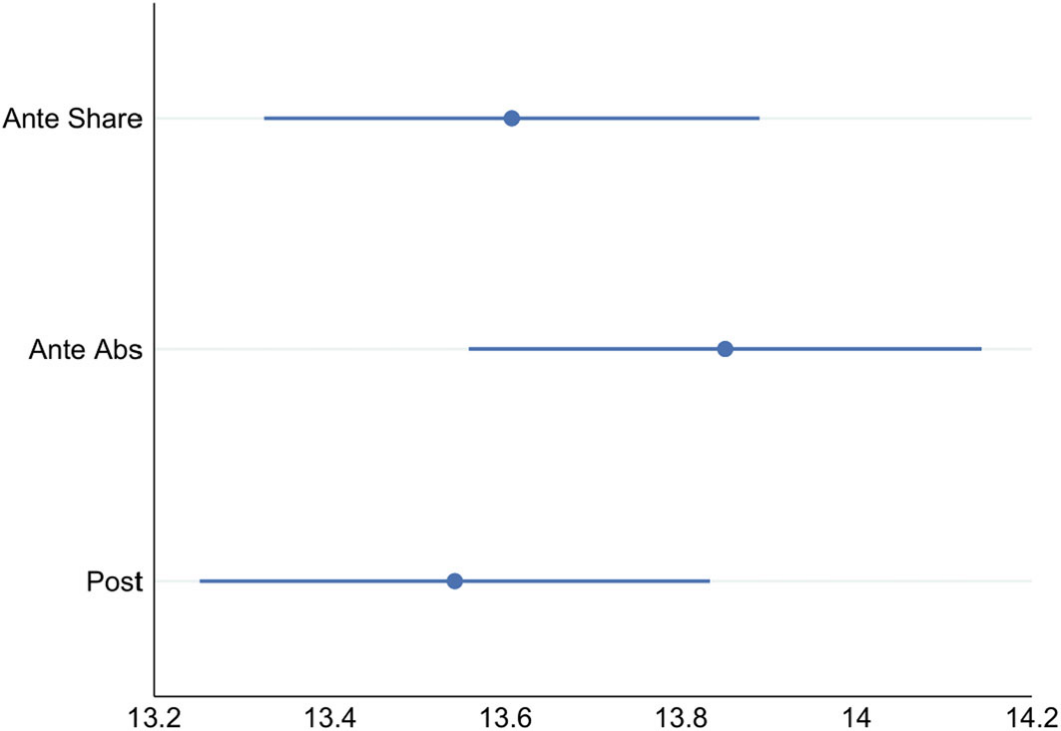
Predicted marginal effects of treatments on effort based on a regression model from Table 2 specification 1. Whiskers indicate 95% confidence intervals. The results reflect the average expected number of correctly solved strings in Part II controlling for other covariates

We find that regardless of the treatment, donors appear to decode more strings in Part II than non-donors. However, this association may be caused by selection: more productive participants may have been more likely to become donors [in line with the literature that higher income is positively associated with donations (e.g., Vesterlund 2006)]. We do not find evidence of an interaction effect between our treatments and donating non-zero amounts (specification 3), suggesting that commitment to giving does not seem to increase productivity.^18^ Finally, we analyze whether some of the increase in productivity of donors may be explained mechanically: to receive the bonus, one needs to meet the income threshold net of donation. Therefore, by committing to give, donors need to earn more to receive the bonus. To account for this effect, we introduce a dummy variable (*Below threshold*) that takes a value of one if given the donation decision and the number of decoded strings in Part I the participant would be below the income threshold. We find no systematic effect of this variable on the number of decoded strings, while the positive effect of the donor status on the number of correctly decoded strings in Part II persists (Table 2, specification 4).

In addition to the number of correctly decoded strings, we recorded how many attempts were made before the correct solution for a string was submitted (Table 5). In general, the participants were fairly accurate with less than 0.4 strings submitted incorrectly on average within 3 min. There is no evidence of treatment differences but donors may have been slightly more accurate even controlling for their initial performance.

Interestingly, while we do not detect any treatment difference in the extended effort, we find differences in the unincentivized question about the perceived effort. In the questionnaire, participants expressed their agreement with the statement that they worked harder because they could donate to the charity on the 7-point Likert scale. Participants in Ante Share tend to agree with the statement more (4.7 out 7, SD 1.9) than participants in Ante Abs (4.2, SD 2.1, t-test $p=0.04$). There is no difference to Post (4.4, SD 2, t-test $p=0.42$). Donors in all treatments expressed stronger agreement than non-donors (5.1, SD 1.68 and 2.6, SD 1.62, t-test $p<0.001$).

**Table 2:** OLS regression

	(1)	(2)	(3)	(4)
Variables	Correct Part II	Correct Part II	Correct Part II	Correct Part II
Ante Abs	0.243	0.219	0.140	0.253
	(0.209)	(0.211)	(0.445)	(0.211)
Post	$-$0.065	$-$0.072	$-$0.240	$-$0.055
	(0.205)	(0.208)	(0.503)	(0.206)
Donation amount	0.913***			
	(0.344)			
Donor (0/1)		0.728***	0.615	0.647**
		(0.244)	(0.457)	(0.252)
Ante Abs:Donor			0.100	
			(0.514)	
Post:Donor			0.219	
			(0.557)	
Correct Part I	0.771***	0.786***	0.786***	0.805***
	(0.039)	(0.037)	(0.038)	(0.038)
Risk-seeking	2.975*	3.567**	3.561**	3.421**
	(1.616)	(1.635)	(1.646)	(1.636)
Entitlement	$-$1.948	$-$2.124	$-$2.128	$-$1.984
	(1.601)	(1.653)	(1.662)	(1.626)
Prosocial	0.016	0.020	0.019	0.014
	(0.036)	(0.037)	(0.037)	(0.037)
Well used money	0.036	0.013	0.015	0.004
	(0.057)	(0.064)	(0.063)	(0.063)
Below threshold (0/1)				0.282
				(0.190)
Constant	3.429***	3.022***	3.099***	2.859***
	(0.600)	(0.567)	(0.683)	(0.567)
Observations	437	437	437	437
R^2^	0.689	0.689	0.689	0.690
F-test (Ante Abs = Post)				
F-statistic	2.17	1.98	0.97	2.19
p-value	0.14	0.16	0.33	0.14

(0/1) indicates dummy variables. The variable *Below threshold* takes the value of 1 if a participant given their performance in Part I and their donation decision would be below the threshold to obtain the bonus

Robust standard errors in parentheses

*** p<0.01, ** p<0.05, * p<0.1

**Result 5:** Contrary to our Hypotheses 2 and 3, there is no significant effect of the commitment to give on effort in Part II. However, there is a positive association between an increase in effort exerted and being a donor.

**Statistical power** A caveat on the statistical power of our study to identify treatment differences is necessary here. The study was designed to detect small to medium effect sizes of Cohen’s *d* of 0.3 (Cohen 2013) with 80% power ($\alpha$ error probability of 0.05) in t-tests and small effect sizes of $f^2=0.025$ in a regression with 3 tested predictors and 8 total predictors.

However, if we consider the difference in effort detected between Ante Share and Post, it is only 0.12 of Cohen’s *d* effectively meaning that effort extended by 95.2% of the two groups overlap (Magnusson 2023). Additionally, for differences in the share of donors between the two Ante treatments, the effect size we observe equals Cohen’s *d* of 0.1. Putting this into perspective, it can be roughly interpreted as 10% of the standard deviation difference between the means (Lakens 2013).

## Discussion and conclusion

In this paper, we study how the timing and the type of donations affect the willingness to donate part of one’s income and the effort extended to generate this income. For this, we conduct an online real-effort experiment with working-age participants from the UK. We opted for a fairly tedious real-effort task to stress the importance of monetary incentives to participants. The novel feature of our experiment is the introduction of a subsistence income constraint, designed as an income threshold and a bonus. This constraint mimics the fact that many real-world labor-leisure and donation choices are influenced by external restrictions such as subsistence income requirements. For instance, people need to earn a minimum amount of money in order to be able to properly take part in their societies’ lives. The existence of the subsistence threshold puts some stress on potential donors when deciding on how much to donate and how hard to work.

If participants face a threshold, the time when a donation decision is made relative to the generation of income becomes relevant. If the decision is made before the generation of income, very generous donors may risk not meeting the threshold. If the decision is made after the generation of income, that is, after the effort was extended, donors may form a feeling of entitlement towards their earnings and give less. Additional proxies allow us to test for the effect of both motives. We find some suggestive, but not robust, evidence that both effects influence giving. However, in our setting, they seem to cancel each other out. Therefore, in our experiment, the timing of the donation decision does not appear to affect giving. Although we chose a tedious task to allow for the entitlement effect, it is feasible that it is context-specific and therefore weaker or stronger for different people outside the stylized environment.

Expressing the donations as an absolute amount and not as a share of future income appears to negatively affect the likelihood of donating and the amount donated. Our results suggest that better performers may donate more, but document no evidence of an additional effect of an advance commitment on giving on their effort. Interestingly, it appears that (unincentivized) measures of prosociality are positively associated with the propensity to donate and donation amounts, but are not related to effort.

In our experiment, participants could donate to a well-known and reputable charitable organization, the British Red Cross, but could not choose the charity themselves. Heterogeneity in their awareness of the organization and their attitudes to its effectiveness may have played a role in their giving and effort. To control for these potential differences, we asked if participants knew the British Red Cross and collected their beliefs about how likely it is that the money would be well spent. To curb potential distrust of online participants towards experimenters delivering on the promise to donate, we repeated in the instructions that participants would receive a confirmation certificate via Prolific shortly after the study (which they did). Given the high share of donors in our study, we believe that the distrust towards the procedure was of little concern.

This study offers avenues for follow-up work to consider possible extensions of the framework. For example, a lot of scientific interest was recently dedicated to pledging, a non-binding commitment to give. Andreoni and Serra-Garcia (2021a) find that (although non-binding) pledges can be effectively used to screen potential donors: for people who make a non-binding pledge, other measures that increase conversion (e.g., sending thank you notes) proved particularly effective in increasing actual donations. The work of Fosgaard and Soetevent (2018) suggests that the firmer the pledge, the more closely the amount donated matches the amount that was pledged. Considering volunteering as opposed to monetary donations, Capra et al. (2022) document that pledges do not lead to an increase in volunteering. Our experiment can offer only limited insights into the issue of reneging and how it would affect the donations because the focus of our study was binding commitments. By offering participants in the Ante treatments a second hypothetical donation decision after income was generated, we documented the intention to renege “positively” (i.e., more people wanted to donate and the amount donated was slightly larger on average). However, the decision is hypothetical, and charitable giving has a high social desirability. Therefore, those results have to be evaluated cautiously.

Another potential extension could relate to the subsistence threshold. In our study, the threshold is the same for all participants and it is exogenous. In future studies, one could further manipulate and refine it. For instance, different people may face different thresholds based on their individual needs. It is also feasible that people are not sure about their threshold in advance (e.g., unexpected medical expenses), and their behavior may influence the probability of a change in the threshold (e.g., working too hard might lead to health issues and thus make medical expenses more likely) thus making the threshold endogenous. This may amplify the perception of risk and influence the willingness to donate in the Ante conditions.

The most compelling implication arising from our findings is that soliciting donations before income is generated and framing the donation as a share of future earnings, rather than fixed absolute amounts, may serve as an effective strategy to enhance charitable giving. Understanding these behavioral nuances in donation decisions can aid policymakers, non-profit organizations, and fundraisers in formulating more effective strategies to promote philanthropy and social welfare.

## Data Availability

Study materials including the original data can be found in the Open Science Framework repository (https://osf.io/pbajr/).

## Additional Tables

**Table 3:** OLS regression

	(1)	(2)	(3)	(4)
	Donation	Donation	Donation	Donation
Variables	amount	amount	amount	amount
Ante Pooled	0.114	0.009		
	(0.086)	(0.033)		
Ante Abs			0.152	$-$0.093***
			(0.093)	(0.035)
Ante Post			$-$0.051	$-$0.056
			(0.093)	(0.039)
Risk-seeking	0.713	0.286	0.766*	0.348
	(0.477)	(0.274)	(0.417)	(0.280)
Entitlement	$-$0.254	0.077	$-$0.256	$-$0.504
	(0.264)	(0.476)	(0.262)	(0.395)
Ante Pooled:Risk-seeking	$-$0.686			
	(0.553)			
Ante Pooled:Entitlement		$-$0.546		
		(0.541)		
Ante Abs:Risk-seeking			$-$1.542***	
			(0.584)	
Ante Post:Risk-seeking			$-$0.048	
			(0.616)	
Ante Abs:Entitlement				0.071
				(0.530)
Ante Post:Entitlement				0.566
				(0.603)
Previous performance	0.021***	0.021***	0.022***	0.022***
	(0.005)	(0.005)	(0.004)	(0.005)
Prosocial	0.028***	0.028***	0.027***	0.027***
	(0.006)	(0.006)	(0.006)	(0.006)
Well used money	0.069***	0.070***	0.067***	0.069***
	(0.009)	(0.009)	(0.009)	(0.009)
Expected pay	$-$0.031**	$-$0.030**	$-$0.032**	$-$0.031**
	(0.014)	(0.014)	(0.014)	(0.014)
Constant	$-$0.580***	$-$0.522***	$-$0.526***	$-$0.468***
	(0.104)	(0.092)	(0.099)	(0.087)
Observations	437	437	437	437
R^2^	0.209	0.208	0.230	0.220

The treatment indicator *Ante Pooled* reports the results of Ante Share and Ante Abs jointly. *Risk-seeking* is measured through the investment in the risky project in the first investment game. *Entitlement* is measured through the difference in investments between the first and second investment games. *Previous performance* refers to the number of correct entries in Part I in the Ante treatments and in Part II in the Post treatment. *Prosociality* and *Well-used money* were reported on 10-point and 7-point scales respectively with higher values indicating higher prosociality and stronger agreement that the money will be well spent

Robust standard errors in parentheses

*** p<0.01, ** p<0.05, * p<0.1

**Table 4:** OLS regression

	(1)	(2)	(3)	(4)
Variables	Correct Part II	Correct Part II	Correct Part II	Correct Part II
Ante Pooled	0.185	0.181	0.326	0.181
	(0.179)	(0.179)	(0.378)	(0.178)
Donation amount	0.876**			
	(0.342)			
Donor (0/1)		0.710***	0.839**	0.636**
		(0.241)	(0.381)	(0.251)
Ante:Donor			$-$0.196	
			(0.430)	
Correct Part I	0.774***	0.788***	0.789***	0.805***
	(0.039)	(0.037)	(0.037)	(0.038)
Risk-seeking	3.142*	3.701**	3.661**	3.590**
	(1.615)	(1.633)	(1.657)	(1.634)
Entitlement	$-$1.971	$-$2.137	$-$2.122	$-$2.015
	(1.602)	(1.651)	(1.656)	(1.627)
Prosocial	0.013	0.016	0.016	0.011
	(0.036)	(0.037)	(0.037)	(0.037)
Well used money	0.036	0.014	0.016	0.005
	(0.057)	(0.063)	(0.063)	(0.063)
Below threshold (0/1)				0.249
				(0.192)
Constant	3.337***	2.941***	2.835***	2.811***
	(0.591)	(0.564)	(0.598)	(0.569)
Observations	437	437	437	437
R^2^	0.688	0.688	0.688	0.689

Replication of the Table 2 with two Ante treatments pooled. (0/1) indicates dummy variables. The variable *Below threshold* takes the value of 1 if a participant, given their performance in Part I and their donation decision, would be below the threshold to obtain the bonus

Robust standard errors in parentheses

*** p<0.01, ** p<0.05, * p<0.1

**Table 5:** OLS regression

	(1)
Variables	Wrong attempts Part II—Part I
Ante Abs	$-$0.047
	(0.094)
Post	$-$0.123
	(0.112)
Donor (0/1)	$-$0.232*
	(0.119)
Correct Part I	0.082***
	(0.017)
Risk-seeking	$-$1.980**
	(0.868)
Entitlement	1.985**
	(0.785)
Prosocial	$-$0.005
	(0.019)
Well used money	$-$0.026
	(0.035)
Constant	$-$0.328
	(0.302)
Observations	437
R^2^	0.106

Difference in the number of wrong attempts in Part II and Part I. A positive number indicates that participants made more incorrect attempts in Part II as compared to Part I

Robust standard errors in parentheses

*** p<0.01, ** p<0.05, * p<0.1

## Footnotes

1 The Founders pledge, for example, allows tech entrepreneurs to make a legally binding regular commitment to donate 2% or more of their potential revenues to a chosen charity (https://www.founderspledge.com/support-our-mission [accessed June 8th 2024]).

2 Further experimental evidence on interplay between the entitlement to an endowment and the specific form of fairness that guides the distribution of the endowment is provided by Cappelen et al. (2007), Cappelen et al. (2013b), Cappelen et al. (2013a).

3 As the task is identical to Part I, participants can estimate how well they will do in Part II based on their performance in Part I.

4 We inform participants about this rule before they choose their donations. It ensures that people cannot donate more than they earn.

5 In the Post treatment we do not differentiate between donations expressed as a share and as an absolute amount given that the income has already been generated.

6 This framework depicts many real-world examples: *i* may need an income net of transfers that is sufficiently high to finance *i*’s minimum living standards. *i* may also run a company, and her income net of transfers may need to be sufficiently high for the company to continue its business. In our experimental setting, *i* simply receives a bonus if her income net of transfers meets a minimum income requirement to represent the value of future earnings.

7 Notice that $\frac{\partial p(y)}{\partial y}\le 0$ (i.e., larger transfers decrease the likelihood that *i* completes the period successfully).

8 $y^{*Treatement}$ is the optimal donation in the corresponding treatment condition. $x_i^{Treatement}$ is effort in the corresponding treatment condition.

9 We recruited 449 participants. Two were excluded from the analysis as they experienced technical problems during the experiment, four were identified as outliers based on their performance in Part I (more than three standard deviations from the mean), six participants revealed to have misunderstood the instructions. We, therefore, perform the analysis on the remaining 97.3% of the sample (437 observations).

10 On average £0.3 from this amount constituted a donation and was transferred directly to the British Red Cross.

11 All reported tests are two-sided.

12 The difference between Ante Share and Post is not significant, $p=0.5$.

13 We additionally explore whether the role of risk-seeking and entitlement differs by treatment and introduce the corresponding interaction effects into our main regression specification (Table 3). Risk-seeking seem to matter differently in Ante Abs, however, there are no systematic differences between the pooled Ante treatments and Post.

14 35 participants (18 in the Ante Share and 17 in the Post treatment) decided to donate 100% of their earnings to a charity thus giving up on the bonus regardless of how hard they worked. These participants on average solved 11.4 strings in Part I, meaning that their initial performance was about average (11.8 strings in the whole sample) and reported to be very prosocial (8.1 out of 10). We excluded these participants from this part of the analysis.

15 The difference between Ante Abs and Ante Share is not statistically significant, $p=0.11$.

16 We only offered a hypothetical donation decision in the Ante treatments to avoid any possible contamination of the main outcome of interest (i.e., the actual donation decision) in Post due to, for instance, a preference for consistency.

17 In this part of the analysis, controlling for the expected pay does not contribute to the explanatory power of the model. Adding this component does not change the magnitude of the effects nor their significance.

18 Pooling the two Ante treatments to test for a motivational effect does not change the results. In a specification otherwise identical to specification 3 in Table 2 the interaction term between the dummy for both Ante treatments and being a donor is $\beta =-0.20, p= 0.65$, see Table 4.
